# Prognostic analysis for Chinese patients with stage I ovarian endometrioid carcinoma

**DOI:** 10.1186/s13048-017-0361-0

**Published:** 2017-09-18

**Authors:** Yu Zhao, Shu Wang, Yi-Min Qu, Yu-Ting Ji, Keng Shen, Jing He Lang

**Affiliations:** 1 Department of Obstetrics and Gynecology, Peking Union Medical College Hospital, Peking Union Medical College & Chinese Academy of Medical Sciences, 1 Shuai Fu Yuan, Dong Cheng District, Beijing, 100730 People’s Republic of China; 2 Department of Gynecology, Women’s Hospital School of Medicine Zhejiang University, Hangzhou, People’s Republic of China; 3 School of Public Health, Peking Union Medical College & Chinese Academy of Medical Sciences, Beijing, People’s Republic of China

**Keywords:** Ovarian endometrioid carcinoma, Stage I disease, Prognosis

## Abstract

**Background:**

This study aimed to identify the clinical and pathological characteristics and the possible prognostic factors for Chinese patients with early-stage ovarian endometrioid carcinoma.

**Methods:**

The present study reviewed the medical records of patients who received initial treatment and a postoperative pathological diagnosis of ovarian endometrioid carcinoma at our center. In all, 78 patients had stage I ovarian endometrioid carcinoma.

**Results:**

In this series, the 5-year overall survival rate and 5-year disease-free survival (DFS) rates of patients with stage I ovarian endometrioid carcinoma was 98.7% and 87.2%, respectively. Univariate analysis showed the factors that influence the DFS rates include menopausal status, FIGO stage, histological grade, lymphadenectomy, cytology of ascites. Multivariate analysis showed that grade 3 and lymphadenectomy were the independent prognostic factors of DFS for Stage I ovarian endometrioid carcinoma (*P* = 0.0259, 0.0276 respectively). However, the coexisting endometriosis, concomitant endometrial disorders, dissection of para-aortic lymph node and more courses of thermotherapy had no influence on DFS. Besides, it was found that 19.3% of patients in this series had synchronous early stage and well-to-moderate differentiated endometrial carcinoma.

**Conclusions:**

Grade 3 and lymphadenectomy were indicated as the independent factors of DFS for stage I patients with ovarian endometrioid carcinoma. The endometrial changes should be considered seriously when fertility-sparing surgery was planned.

## Background

Epithelial ovarian carcinoma remains the most lethal of gynecologic malignancies. Numerous studies have revealed the various histological subtypes of ovarian cancers might have distinguishing origination and mechanism of development, and divergent clinical and pathological characters and different prognosis as well [1]. According to literature, it was believed that ovarian endometrioid carcinoma (OEC) accounts for 16–25% of all epithelial ovarian cancers [2]. Compared with patients with high-grade serous carcinoma, a higher percentage of patients who are diagnosed with OEC are at the early stage, and the prognosis of this series of patients is relatively better. It is currently believed that patients with International Federation of Gynecology and Obstetrics (FIGO) stage I ovarian endometrioid carcinoma have a good overall prognosis and low rates of cancer recurrence. However, a uniform and clear consensus has not been reached regarding the influence of clinical-pathological features and the extent of surgery on the prognosis of these patients. Therefore, the present study summarized the clinical and prognostic data of Chinese women with stage I OEC who received initial treatment in single institution, in an attempt to identify the prognostic factors for patients in this seris.

## Methods

The present study collected and examined the medical records of patients who received initial treatment and a postoperative pathological diagnosis of OEC at the Department of Obstetrics and Gynecology, Peking Union Medical College Hospital between January 2000 and January 2012. The 188 patients were subjected to surgical-pathologic restaging based on the FIGO staging guidelines for ovarian cancer (2013). In all, 78 patients were found to have FIGO stage I tumor, occupying 41.5% of all OEC during the same period at our center. We collected and statistically analyzed the clinical and pathological data from the 78 patients with stage I OEC.

Here, we defined EOC with concurrent endometriosis as the presence of ovarian cancer and endometriosis identified histologically in the same ovary, the presence of endometriosis in one ovary and of ovarian cancer in the contralateral ovary, or the presence of ovarian cancer and extraovarian pelvic endometriosis (eg, peritoneal endometriosis). Besides, synchronous tumors of the ovary and endometrium were found and analyzed in this series. The criteria of Young and Scully [3] were used for interpretation of synchronous primary tumors of both organs or of metastasis from one organ to the other. When the pathologic study reveals similar types, the differentiation between the 2 separate primary cancers or 1 single advanced cancer with metastasis is much more difficult. Herein, we apply standardized criteria to differentiate the 2 synchronous cancers, rather than 1 cancer metastases [(1) both tumors are confined to primary sites; (2) no direct extension between the tumors; (3) no lymphovascular tumor emboli; (4) no or only superficial myometrial invasion of the endometrial lesion; and (5) no distant metastasis] [4, 5].

In the present study, the patient follow**-**up period ended in March 2017. Disease-free survival (DFS) was defined as the time interval from the date of primary surgery to the date of disease progression and/or recurrence. Overall survival (OS) was defined in months as the date of the primary surgery to the date of death or censoring at the date of last contact. Both OS and DFS were examined. Because the number of deceased cases was too small to reach the significance in Cox model (*n* = 4), only the survival analysis of DFS was performed in this study.

The clinical and prognostic data of the patients were subjected to statistical analysis using the IBM SPSS20 software to screen for the relevant differential information. Survival comparisons were obtained using the log rank test in an unadjusted Kaplan-Meier model. Cox proportional hazards regression was used for multivariate analysis. Receiver Operating Characteristic (ROC) curve was constructed to define the optimal cutoff value for stratifying and grouping. *P* values less than or equal to 0.05 were considered statistically significant.

## Results

In all, 78 patients with stage I OEC were examined in the present study. The clinical and pathological characteristics of the 78 patients were summarized in Tables 1 and 2. At diagnosis, the mean age (±SD) was 48.37 ± 13.29 years, and 60.3% of patients were premenopausal. Respectively 15.4% and 23.1% of patients have never been pregnant and had no child. The common symptoms at initial presentation were sequentially palpable mass, abdominal pain, incidental finding, irregular menstruation and postmenopausal bleeding. The normal preoperative serum value of cancer antigen 125 (Ca125) was seen in 11.5% of patients. The distribution of FIGO stage was 28.2% of Stage Ia, 5.1% of Stage Ib and 66.7% of Stage Ic.

**Table 1:** Clinical and morphological characteristics of patients with stage I OEC

	EAC
Total Number	78
Age(years)	
Mean ± SD	48.37 ± 13.29
Range	24–79
Menopausal status	
Post-menopause	31 (39.7%)
Pre-menopause	47 (60.3%)
Gravid (times)	
0	12 (15.4%)
> =1	66 (84.6%)
Parity (times)	
0	18 (23.1%)
> =1	60 (76.9%)
Symptoms	
Palpable mass	23 (29.5%)
Abdominal pain	17 (21.8%)
Incidental finding	17 (21.8%)
Irregular menstruation	14 (17.9%)
Postmenopausal bleeding	8 (10.3%)
Abdominal distension	6 (7.7%)
Pre-operative Ca125 value (U/ml)	
Median(IQR)	111.5 (58.0, 573.0)
Range	5.2–7600
Ca125 in normal range	
Yes	9 (11.5%)
No	69 (88.5%)
Personal history	
Breast cancer	2 (2.6%)
HT	11 (14.1%)
DM	4 (5.1%)
History of surgical sterilization	4 (5.2%)
TAH	2 (2.6%)
Tube ligation	2 (2.6%)
FIGO Stage^a^	
IA	22 (28.2%)
IB	4 (5.1%)
IC	52 (66.7%)

*Abbreviation*: *OEC* ovarian endometrioid carcinoma, *IQR* InterQuartile Range, *HT* hypertension, *DM* diabetic mellitus, *TAH* total trans-abdominal hysterectomy

^a^according to the classification system of FIGO staging (2013 version)

**Table 2:** Pathological characters and treatments of patients with stage I OEC

	EOC
Total number	78
Tumor size(cm)	
Median [IQR]	8 (6,12)
Range	2.0–25.0
Side of ovarian tumor	
Unilateral	63 (80.8%)
Bilateral	15 (19.2%)
Ascites^b^	
Positive	8 (10.3%)
Negative	70 (89.7%)
Grade	
1	35 (44.9%)
2	26 (33.3%)
3	17 (21.8%)
Mixed histology^c^	
Yes	5 (6.4%)
No	73 (93.6%)
EM associated	
Yes	23 (29.5%)
No	55 (70.5%)
Endometrial disorders^f^	
Yes	21 (26.9%)
No	57 (51.1%)
ER presentation^e^	
Number	37
Positive	25 (67.6%)
Negative	12 (32.4%)
PR presentation^e^	
Number	37
Positive	29 (78.4%)
Negative	8 (21.6%)
Residual disease	
No or <1 cm	77 (98.7%)
> 1 cm	1 (1.3%)
Lymphadenectomy	
Yes	72 (97.4%)
No	6 (2.6%)
Para-aortic lymphadenectomy^a^	
Number	72
Yes	45 (62.5%)
No	27 (37.5%)
Numbers of lymph nodes dissected	
Number	72
Median(IQR, number)	18 [11,27]
Range (number)	2–48
Chemotherapy	
Yes	70 (89.8%)
No	5 (6.4%)
Unclear	3 (3.8%)
Chemotherapy regimen	
Number	70
Platinum based	68 (97.1%)
Others	2 (2.9%)
Platinum chemotherapy cycle	
Number	68
< 4	21 (30.9%)
> =4	47 (69.1%)
Platinum-resistance	
Number	68
Yes	3 (4.3%)
No	65 (95.7%)

*Abbreviation*: *EM* endometriosis, *ER* estrogen receptor, *PR* progesterone receptor

^a^including dissection of common iliac lymph node and para-aortic lymph node

^b^findings of malignant cells in ascites or peritoneal washing

^c^including endometrioid carcinoma mixed with components of serous or clear cell subtypes

^d^including 15 cases of endometrial carcinoma (19.2%), one of endometrial hyperplasia (1.3%), and 5 of endometrial polyps (6.4%)

^e^according to the retrospectively reviewing the results of immunohistogical staining of ER and PR

The pathological examination showed that unilateral tumor occurred in 80.8% of patients, and positive cytology of ascites or peritoneal washing was indicated in 10.3% of patients. Besides, coexisting with endometriosis and synchronous endometrial disorder were confirmed in respectively 29.5% and 26.9% of them, and the latter group included 15 cases of endometrial carcinoma (19.2%), one of endometrial hyperplasia (1.3%), and 5 of endometrial polyps (6.4%). The histological grading was shown as 44.9% of well-differentiation, 33.3% of moderate and 21.8% of poor-differentiation. Of 37 cases whose specimens were examined by immune-staining, respective 67.6% of specimens presented as ER positive and 78.4% as PR positive results.

In this series, 70 patients have received comprehensive staging surgery, and 62.5% of whom were undertaken the dissection of para-aortic lymph node identified by pathology. The median number of dissected lymph node was reported as 18. As shown in Table 2, 70 out of the 78 patients (89.7%) received postoperative chemotherapy. 97.1% (68/70) received platinum-based combination chemo-regimen; 30.9% of whom received no more than 3 courses (mean ± SD, 2.8 ± 0.5 cycles) and 69.1% received 4 courses or more (mean ± SD, 5.8 ± 1.2 cycles). Five patients (6.4%, 5/78) who did not receive postoperative chemotherapy, 4 were diagnosed with stage Ia cancer, and 1 was diagnosed with stage Ic cancer. During the follow-up period, 3 patients developed chemo-resistance to platinum**-**based regimens.

Overall, synchronous endometrial carcinoma was documented in 19.2% of Stage I patients (15/78), which ratio was higher than that in patients at all stage during the same period (9.6%, 18/188) [3]. Interestingly, synchronous tumors of the ovary and endometrium were of identical histological grade in 73.3% (11/15) of cases (Table 3). All of ovarian cancers and synchronous endometrial cancers were grade 1–2 subtypes of histology. And 86.7% of synchronous endometrial cancers were at FIGO stage Ia.

**Table 3:** Histological grades of ovarian and synchronous endometrial cancer (*n* = 15)

	Endometrial cancer			
Ovarian cancer	Total	G1	G2	G3
G1	11	9^a^	2 (1^a^/1^b^)	0
G2	4	2 (1^a^/1^b^)	2^a^	0
G3	0	0	0	0

^a^the synchronous endometrial carcinoma was classified as the FIGO stage Ia (2009)

^b^the synchronous endometrial carcinoma was classified as the FIGO stage Ib (2009)

The median follow-up time was 74.5 [IQR,(56,117)] months. During the follow-up period, 4 patients (5.1%) died, and 17 patients (21.8%) experienced relapses. The median time interval from surgery to recurrence was 39 months [IQR,(26,63)]. And The median time (IQR) of DFS was 67.5 [IQR,(36,101)]. The 5-year OS rate and 5-year DFS rate were respectively 98.7% and 87.2%.

Receiver Operating Characteristic (ROC) curve constructed to stratifying the continuous variable including the age of onset, tumor size, the number of lymph nodes resected and the courses of platinum regimen received. As the results, the optimal cutoff value was defined as 49.5 years for age, 9.5 cm for tumor size, 3.5 times for chemotherapy courses and 17.0 for lymph node dissected (seen in supplement material).

**Fig. 1:**
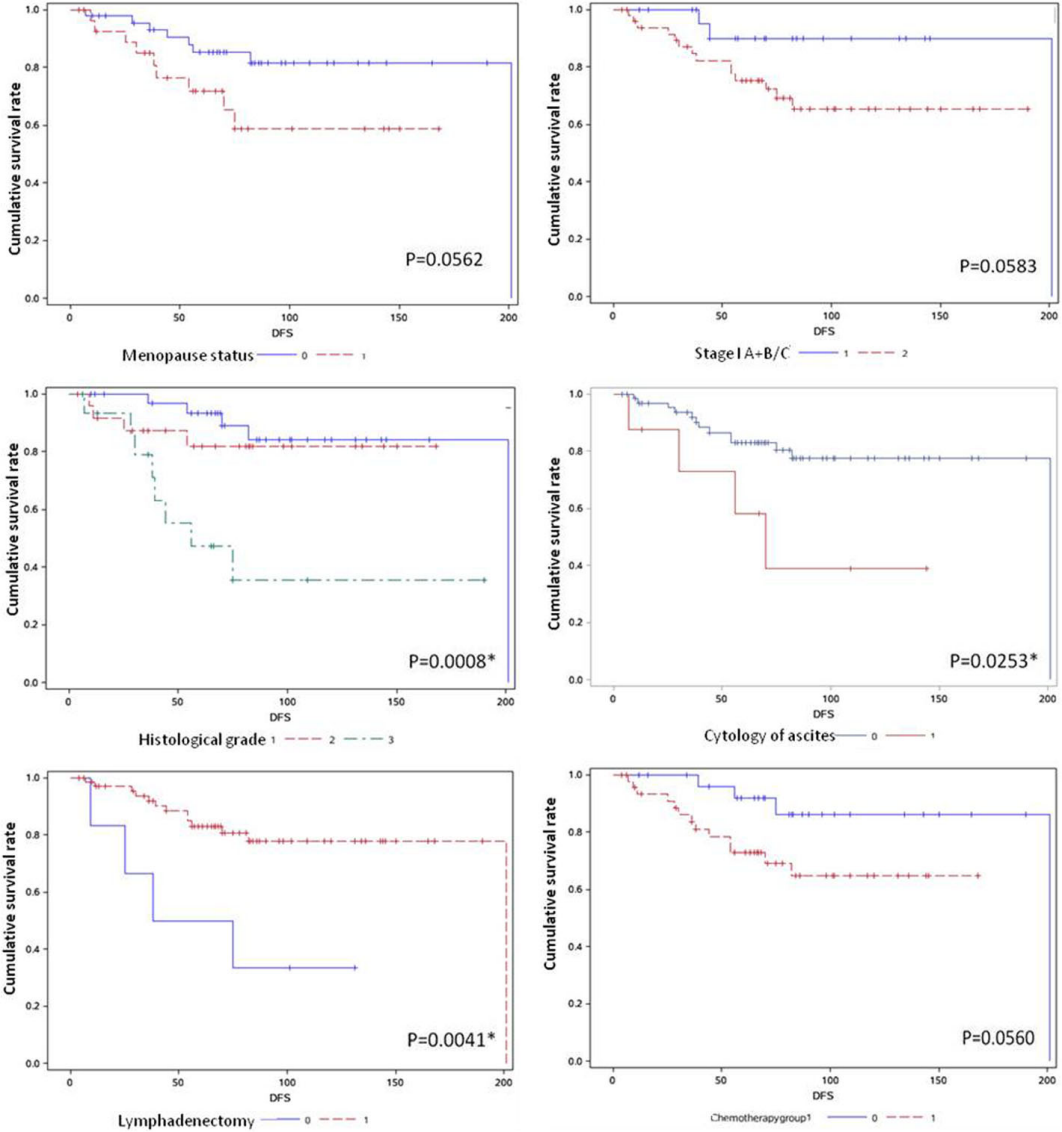
A comparison of DFS in Stage I OEC patients with different variables. Kaplan-Meier survival curves showing the effects of menopausal status (top left), FIGO stage (top right), histological grade (middle left), cytology of ascites (middle right), lymphadenectomy (bottom left), and chemotherapy course group (bottom right)

Patient DFS data were subjected to a univariate analysis using the Kaplan-Meier method (Table 4, Fig. 1). The results showed that the factors that influenced the DFS of patients with stage I OEC included histological grade (*p* = 0.0008), lymphadenectomy (*p* = 0.0041) and cytology of ascites (*p* = 0.0253). The patients at premenopausal status and at Stage Ia-b had less possibility of relapse, when compared with ones post menopause status and at Stage Ic, but the difference did not reach statistical difference in this series (*p* = 0.0526, 0.0583). In contrast, the DFS was not affected by age, being nulliparous or with no child, Ca125 level, tumor size and laterality, whether complicating with hypertension, whether the histology of ovarian cancer was mixed with serous or clear cell component, whether coexisting with endometriosis or endometrial disorders, whether para-aortic lymphadenectomy was performed, the numbers of lymph node resected and the courses of platinum based chemotherapy (all of *p* > 0.05). Besides, of 37 patients with immunostaining examination, the expression of ER or PR was shown no association with DFS of patients with Stage I OEC.

**Table 4:** Univariate analysis of DFS among patients with stage I OEC

Variable	Category	N(%)	DFS(Median(IQR))	DFS rate(%)	P
Age y	<50	45 (57.7%)	75.0 (54.0,102.0)	86.7%	0.2563
	> = 50	33 (42.3%)	56.0 (30.0,81.0)	75.76	
Menopausal status	Pre	47 (60.3%)	82.0 (44.0,109.0)	82.98	0.0526
	Post	31 (39.7%)	56.0 (25.0,78.0)	70.97	
Gravidity	0	12 (15.38)	106.5 (63,143)	83.33	0.2028
	> = 1	66 (84.62)	65.5 (36,90)	77.27	
Parity	0	18 (23.08)	85 (63,131)	83.33	0.1870
	> = 1	60 (76.92)	65 (35,94)	76.67	
CA125 normal	Yes	9 (11.5%)	70 (65 ~ 102)	88.89	0.3948
	No	69 (88.5%)	67 (34 ~ 98)	76.81	
Hypertension	Yes	11 (14.1%)	57 (12 ~ 81)	81.82	0.8304
	No	67 (85.90%)	70 (38 ~ 102)	77.61	
Tumor side	Unilateral	63 (80.77%)	67 (30 ~ 101)	76.19	0.4040
	Bilateral	15 (19.23%)	71 (38 ~ 102)	86.67	
Tumor size	<10	45 (57.69%)	75 (44 ~ 102)	77.78	0.4971
	≥10	33 (42.31%)	56 (16 ~ 84)	78.79	
Endometriosis	Yes	23 (29.5%)	66 (36 ~ 90)	86.96	0.3539
	No	55 (70.5%)	69 (34 ~ 102)	74.55	
Endometrial disorders	Yes	21 (26.9%)	65 (12 ~ 102)	85.71	0.2406
	No	57 (73.1%)	68 (38 ~ 98)	75.44	
Stage	IA + IB	26 (33.3%)	67.0 (39.0,109.0)	88.46	0.0583
	IC	52 (66.7%)	67.5 (29.5,99.5)	73.08	
Mixed histology	No	73 (93.59)	69 (36,102)	79.45	0.1295
	Yes	5 (6.419)	38 (36,65)	60	
Grade	G1	35 (44.8%)	82 (59, 117)	85.71	0.0008*
	G2	26 (33.3%)	64 (29, 98)	84.62	
	G3	17 (21.8%)	39 (28, 66)	52.94	
Cytology of ascites	Negative	70 (89.74)	68.5 [36,101]	81.43	0.0253*
	Positive	8 (10.26)	61.5 [21.5,89.5]	50	
ER	Negative	12 (32.43)	73 [47,102]	83.33	0.7491
	Positive	25 (67.57)	66 [44,86]	80	
PR	Negative	8 (21.62)	68 [49.5,88.5]	87.5	0.6476
	Positive	29 (78.38)	67 [44,90]	79.31	
Lymphadenectomy	Yes	71 (91.0%)	68 (36,102)	81.69	0.0041*
	No	7 (9.0%)	38 (9, 101)	42.86	
Para-aotic Lymphadenectomy	No	27 (37.5)	71 [44,131]	77.78	0.8075
	Yes	45 (62.5)	63 [30,90]	82.22	
Numbers of dissected Lymphadenectomy	<17	34 (43.59)	72.5 [39,131]	76.47	0.8693
	> = 17	38 (48.72)	64 [29,84]	84.21	
Chemotherapygroup	<4	21 (30.9%)	82 (62–121.5)	90.50	0.056
	≥4	47 (69.1%)	65 (30 ~ 101)	72.34	

*P* values were cultivated by Kaplan-Meier analysis

Multivariate Cox regression analysis showed histology grade 3 and whether performing lymphadenectomy to be independent predictors of DFS (HR 4.88, *p* = 0.0259; HR 0.18, *p* = 0.0276), but did not find menopausal status, FIGO stage, cytology of ascites and courses of chemotherapy to be significant predictors (Table 5).

**Table 5:** The multivariate analysis of DFS among patients with stage I OEC

Variable	β	P	HR(95% CI)
Menopause	0.33464	0.6072	1.4 (0.39 ~ 5.01)
Stage	0.15452	0.856	1.17 (0.22 ~ 6.2)
Grade2–1	0.58496	0.4369	1.8 (0.41 ~ 7.84)
Grade3–1	1.58441	0.0259*	4.88 (1.21 ~ 19.66)
Lymphadenectomy	−1.737	0.0276*	0.18 (0.04 ~ 0.83)
Cytology of ascites	0.55961	0.4701	1.75 (0.38 ~ 7.99)
Chemotherapy cycle	1.32926	0.0598	3.78 (0.95 ~ 15.08)

*P* values were cultivated by Cox regression analysis. The overall test of the above model showed the model was significance, *p* = 0.0068

## Discussion

The 78 patients with stage I OEC who were included in the present study accounted for 41.5% of all stage patients with this subtypes of tumor during the same period, which is consistent with the previously reported range of 34–47% and is significantly higher than the percentage of stage I patients with ovarian serous carcinoma (9–12%) [2, 6–9]. The above finding indicates that a considerable proportion of patients with OEC are diagnosed at an early stage. Primary symptoms experienced by the group of patients with OEC included a palpable pelvic mass (29.5%), abdominal pain (21.8%) and abnormal vaginal bleeding (including menstrual abnormalities and postmenopausal bleeding, 28.2%). Regarding specific early onset symptoms might be helpful to the early detection and diagnosis of this subtype of ovarian cancer.

The present study found that the average age at onset was 48 years in this group, which is younger than stage I/II patients reported by Kumar et al. (the average age = 52 years) [10]. Besides, 60.3% of patients were at premenopausal status and 23.1% of them have no child in the present study, which findings raise the question if more patients in this group had desire of preserving fertility. Additionally, 78.2% of the patients in this study had G1–2 tumor, 33.3% of them were at FIGO Ia and Ib stage, and 80.8% of them had unilateral tumor. According to the 2016 National Comprehensive Cancer Network (NCCN) guidelines, the patients of OEC with G1–2 tumor and at Stage Ia or Ib could considered to be performed fertility-sparing comprehensive staging surgery [11]. Our survival data showed that 9 patients whose situation were accordance to the fertility-sparing criteria mentioned above (premenopausal, G1–2 tumor, FIGO stage Ia-Ib) had 5-year DFS rate of 100%; and of them, the only one who had relapse tumor at the end of this study had the disease free interval of 201 months after primary surgery.

However, our data also indicated 19.3% of patients with Stage I OEC had synchronous endometrial cancer, which situation should be seriously taken into account particularly for patients with the desire of receiving the fertility-sparing surgery. The comprehensive evaluation of endometrial might be necessary. However, the best part is that all concomitant endometrial carcinoma in this series were with G1–2 tumors and at FIGO Stage Ia-Ib, and the survival of patients with synchronous endometrial carcinoma showed no difference with ones without, which was accordance to the results of Kelemen LE et al. [12] Although we use the standard of synchronous tumor widely accepted by clinicians, we could not actually distinguish the difference between the synchronous early Stage ovarian and endometrial cancer and metastasis tumor. The relative molecular studies performed by our team are ongoing, we hope the upcoming result help us to make this question clear and resolve this dilemma.

On the contrary, Grade 3 was indicated as the independent factor of DFS, which group of patients had 4.88 times of risk of relapse (*p* = 0.0259) and 5-year DFS rate of 64.7%.Besides, the patients at postmenopausal status had higher possibility of relapse when compared with women at premenopausal status (5-year DFS, the corresponding 5-year survival rates were 84.5% and 71.6%, but with no statistical significance (*p* = 0.0526). respectively. The nulliparity also showed no relationship with the risk of DFS according to this series of data.

Moreover, our results revealed that the DFS was not affected by age, being nulliparous or with no child, Ca125 level, tumor size and laterality, whether complicating with hypertension, whether the histology of ovarian cancer was mixed with serous or clear cell component, whether coexisting with endometriosis or endometrial disorders; which was somewhat divergent with the previous similar reports [13].

The 2016 National Comprehensive Cancer Network (NCCN) guidelines listed hormone therapy as a postoperative adjuvant treatment option for histologic grade 1 OEC and low-grade serous carcinoma; examples of hormone therapy include medications such as aromatase inhibitors, leuprolide acetate, and tamoxifen [11]. Our data showed respective 67.6% of ovarian tumor presented as ER positive and 78.4% as PR positive staining, which provided the evidence for hormone treatment, although we had no related experience of clinical practice of hormone treatment. Rambau P et al. reported that Expression of ER and PR were significantly associated with longer ovarian cancer specific survival, but no association was found in this study [14].

It has been previously reported that the 5-year postoperative survival rate of patients with stage I OEC exceeds 90%. Chan et al. analyzed the prognosis of 1718 patients with stage I OEC from the Surveillance, Epidemiology and End Results (SEER) database. The results showed that the 5-year OS rate was 92.7%, while the OS rates of patients with stage Ia, Ib, and Ic OEC were 94.8%, 91.2%, and 89.2%, respectively. The survival rate of patients with stage I OEC was higher than the survival rates of patients with stage I serous carcinoma and clear cell carcinoma [9]. In the present study, the 5-year OS rate of patients with stage I OEC was 98.7%, while the survival rates of patients with stage Ia, Ib, and Ic OEC were 100%, 100%, and 97.1%, respectively. The results of the present study were similar to those published in previous reports.

In addition, the present study showed that the 5-year DFS rate of the group of patients with stage I OEC was 83.3%, and the 5-year DFS rates of patients with stage IA/IB and IC OEC were 92.3% and 78.8% respectively, but no statistical difference (*p* = 0.0583). However, our data showed that cytology of ascites or peritoneal washing was the risk factor of DFS (*p* = 0.0253), which group was at FIGO Stage Ic3 according to 2014 classification system; the 5-year DFS of rate of patients in this group was only 62.5%, but multivariate analysis indicated that was not the independent prognostic factor of DFS. Kumar et al. reported that the 5-year DFS rates of patients with stage IA/IB, stage IC1 and stage IC2/IC3 OEC were 95%, 84%, and 74%, respectively [10]. In the study conducted by Storey et al., the 5-year DFS rate of patients with stage I OEC was 79%, which was higher than the 5-year DFS rate of patients with stage I serous carcinoma (70%), but no significant difference [6]. The above results indicate that the overall and disease-free prognosis of Stage Ia/b OEC are fairly good and that the tumor recurrence rate and mortality rate are lower in early-stage OEC compared with early-stage serous carcinoma.

This study also showed that lymphadenectomy was the independent protective factor for postoperative relapse for Stage I OEC (*p* = 0.0041), but the number of dissected lymph nodes was not. Theoretically, surgical resection of lymph nodes is conducive to preventing tumor micrometastasis in patients with early-stage cancers. It has been reported in the literature that lymph node dissection reduces the probability of recurrence in patients with stage Ic or G2/G3 ovarian cancer, but had no effect on patients with stage IA/IB G1 ovarian cancer [15–17]. However, in Maggioni et al.’s study, 268 patients with early-stage ovarian cancer were randomly assigned to undergo lymphadenectomy and lymph node sampling and the results showed no significant difference in postoperative survival between the patients with or without being underwent lymphadenectomy [18]. And Zhou et al. have conducted a meta-analysis and showed that systematic lymphadenectomy improved the OS for early-stage ovarian cancer patients, but not DFS [19]. The results of the latest larger-scale clinical study have verified lymphadenectomy associated with a survival advantage for those with endometrioid carcinoma [20].

Furthermore, there is no unified opinion regarding whether para-aortic lymphadenectomy is necessary. In the present study, dissection of para-aortic lymph node showed no relation with DFS (*p* = 0.8075). Oshita et al. have demonstrated that para-aortic lymphadenectomy had no significant effect on OS or DFS in patients with stage pT1 ovarian cancer [21]. Many scholars believe that in order to reduce postoperative cancer recurrence, high-level lymphadenectomy should be actively performed in patients with stage Ic ovarian cancer and poorly differentiated cancer identified by intra-operative pathologic examinations. However, these previously published studies did not investigate various histological subtypes of epithelial ovarian cancers separately. Therefore, additional in-depth studies in this aspect are much needed in future.

In addition, the present study showed that resistance to platinum-based drugs rarely developed in patients with stage I OEC. Among the 68 patients who received postoperative platinum-based combination chemotherapy, only 3 patients (4.3%) developed resistance to platinum-based chemotherapy drugs. And there was no difference found for the DFS of patients with less than 4 cycles of platinum-based chemotherapy and more than 4 cycles. It needs more efforts to investigate the optimal cycles of postoperative chemotherapy for Stage I OEC patients with variable prognostic risk factors.

## Conclusion

At the time of onset, a large percentage of patients with OEC have stage I cancers. The overall prognosis of patients with OEC is relatively good. The independent prognostic factors for DFS are shown as the degree of tumor differentiation and whether the patients underwent lymphadenectomy. In contrast, the DFS was not affected by age, being nulliparous or with no child, Ca125 level, tumor size and laterality, whether complicating with hypertension, whether the histology of ovarian cancer was mixed with serous or clear cell component, whether coexisting with endometriosis or endometrial disorders, whether para-aortic lymphadenectomy was performed, the numbers of lymph node resected and the courses of platinum based chemotherapy. The present study was a single-center retrospective study, and the number of medical cases was limited when they were stratified based on certain parameters, which might all affect the analysis results. Therefore, we expect more high-quality clinical studies focusing on early-stage OEC, which definitely will improve our understanding of this histological subtype of epithelial ovarian cancer and be conducive to achieving a better therapeutic decision-making and improved prognosis for patients in this group.

## Abbreviations

CA125: Cancer antigen 125
DFS: Disease free survival
FIGO: International Federation of Gynecology and Obstetrics
NCCN: National Comprehensive Cancer Network
OEC: Ovarian endometrioid carcinoma
OS: Overall survival
ROC: Receiver Operating Characteristic

## References

[1] Kurman RJ, Shih IM (2008). Pathogenesis of ovarian cancer: lessons from morphology and molecular biology and their clinical implications. Int J Gynecol Pathol.

[2] Storey DJ, Rush R, Stewart M, Rye T, Al-Nafussi A, Williams AR (2008). Endometrioid epithelial ovarian cancer: 20 years of prospectively collected data from a single center. Cancer.

[3] Young RH, Scully RE, Kurman RJ (1994). Metastatic tumors of the ovary. Blaustein’s pathology of the female genital tract.

[4] Yang YH, Chen RJ, Lin MC, Cheng SP, Chang TC (2010). Synchronous primary ovarian and endometrial cancer with a fair prognosis in a young woman. Taiwan J Obstet Gynecol.

[5] Ree YS, Cho SH, Kim SR, Cho SH, Kim KT, Park MH (2003). Synchronous primary endometrial and ovarian cancer with three different histologic patterns: a case report. Int J Gynecol Cancer.

[6] Wang S, Qiu L, Lang JH, Shen K, Huang HF, Pan LY (2013). Prognostic analysis of endometrioid epithelial ovarian cancer with or without endometriosis: A 12-year cohort study of Chinese patients. Am J Obstet Gynecol.

[7] Grosso G, Raspagliesi F, Baiocchi G, Di Re E, Colavita M, Cobellis L (1998). Endometrioid carcinoma of the ovary: a retrospective analysis of 106 cases. Tumori.

[8] Chan JK, Teoh D, Hu JM (2008). Do clear cell ovarian carcinomas have poorer prognosis compared to other epithelial cell types? A study of 1411 clear cell ovarian cancers. Gynecol Oncol.

[9] Kumar A, Le N, Tinker AV, Santos JL, Parsons C, Hoskins PJ (2014). Early-Stage Endometrioid Ovarian Carcinoma Population-Based Outcomes in British Columbia. Int J Gynecol Cancer.

[10] Sieh W, Köbel M, Longacre TA, Bowtell DD, de Fazio A (2013). Hormone receptor expression and ovarian cancer survival: an Ovarian Tumor Tissue Analysis consortium study. Lancet Oncol.

[11] Morgan RJ, Armstrong DK, Alvarez RD, Bakkum-Gamez JN, Behbakht K, Chen LM (2016). Ovarian Cancer, Version 1.2016, NCCN Clinical Practice Guidelines in Oncology. J Natl Compr Cancer Netw.

[12] Kelemen LE, Rambau PF, Koziak JM, Steed H, Köbel M (2017). Synchronous endometrial and ovarian carcinomas: predictors of risk and associations with survival and tumor expression profiles. Cancer Causes Control.

[13] Minlikeeva AN, Freudenheim JL, Cannioto RA, Szender JB, Eng KH, Modugno F (2017). History of hypertension, heart disease, and diabetes and ovarian cancer patient survival: evidence from the ovarian cancer association consortium. Cancer Causes Control.

[14] Rambau P, Kelemen LE, Steed H, Quan ML, Ghatage P, Köbel M. Association of Hormone Receptor Expression with Survival in Ovarian Endometrioid Carcinoma: Biological Validation and Clinical Implications. Int J Mol Sci. 2017;18(3): pii: E515) 10.3390/ijms18030515. 10.3390/ijms18030515 PMC5372531 28264438

[15] Lang JH (1994). Lymph node metastasis in stage I ovarian carcinoma. Chin Med J.

[16] Suzuki M, Ohwada M, Yamada T, Kohno T, Sekiguchi I, Sato I (2000). Lymph node metastasis in stage I epithelial ovarian cancer. Gynecol Oncol.

[17] Tognon G, Carnazza M, Ragnoli M, Calza S, Ferrari F, Gambino A (2013). Prognostic factors in early-stage ovarian cancer. Ecancermedicalscience.

[18] Maggioni A, Benedetti PP, Dell'Anna T, Landoni F, Lissoni A, Pellegrino A (2006). Randomised study of systematic lymphadenectomy in patients with epithelial ovarian cancer macroscopically confined to the pelvis. Br J Cancer.

[19] Zhou J, Shan G, Chen Y. The effect of lymphadenectomy on survival and recurrence in patients with ovarian cancer: a systematic review and meta-analysis. Jpn J Clin Oncol. 2016;46(8):718-26. 10.1093/jjco/hyw068 27272175

[20] Nasioudis D, Chapman-Davis E, Witkin SS, Holcomb K (2017). Prognostic significance of lymphadenectomy and prevalence of lymph node metastasis in clinically-apparent stage I endometrioid and mucinous ovarian carcinoma. Gynecol Oncol.

[21] Oshita T, Itamochi H, Nishimura R, Numa F, Takehara K, Hiura M (2013). Clinical impact of systematic pelvic and para-aortic lymphadenectomy for pT1 and pT2 ovarian cancer: a retrospective survey by the Sankai Gynecology Study Group. Int J Clin Oncol.

